# Antibacterial Activities of Phytofabricated ZnO and CuO NPs by *Mentha pulegium* Leaf/Flower Mixture Extract against Antibiotic Resistant Bacteria

**DOI:** 10.34172/apb.2021.057

**Published:** 2020-08-05

**Authors:** Mehran Alavi, Saeed Dehestaniathar, Shadieh Mohammadi, Afshin Maleki, Naser Karimi

**Affiliations:** ^1^Environmental Health Research Center, Research Institute for Health Development, Kurdistan University of Medical Sciences, Sanandaj, Iran.; ^2^Nanobiotechnology Laboratory, Biology Department, Faculty of Science, Razi University, Kermanshah, Iran.

**Keywords:** Phytosynthesis, Metal oxide NPs, Mentha pulegium, Antibiotic resistance, Antibacterial activities

## Abstract

***Purpose:*** In this study, leaf/flower aqueous extract of medicinal plant species *Mentha pulegium* was used to synthesize ZnO and CuO nanoparticles (NPs) as a cost-effective, one-step, and eco-friendly method.

***Methods:*** Physicochemical properties of both metal oxide NPs (MONPs) were determined by UV-Vis spectroscopy, X-ray diffraction (XRD), Fourier-transform infrared (FTIR) spectroscopy, scanning electron microscope (SEM) and energy dispersive X-ray (EDX) techniques.

***Results:*** Phytofabricated ZnONPs and CuNPs illustrated 65.02±7.55 and 26.92±4.7 nm with antibacterial activities against antibiotic-resistant *Escherichia coli* and *Staphylococcus aureus*. Higher antibacterial activities were observed for CuONPs compared with ZnONPs.

***Conclusion:*** Large surface area and more reactivity resulted from smaller size as well as higher production of reactive oxygen species (ROS) were considered to antibacterial efficiency of CuONPs against antibiotic-resistant *E. coli* and *S. aureus*.

## Introduction

Infectious diseases resulted from antibiotic-resistant microorganisms specifically multidrug-resistant (MDR) bacteria are increasing owing to the inefficiency of conventional antibiotics.^1^ Several mechanisms including higher expression of efflux pump and antibiotic degradable enzymes are recognized for this ability in bacteria.^2^ In recent years, nanotechnology has illustrated the appropriate antibacterial capacities of metal nanoparticles (MNPs)/metal oxide NPs (MONPs) compared to their bulk materials and common antibiotics.^3^ These abilities can be resulted from unique properties such as higher aspect ratio and surface area to volume ratio of these nanomaterials (NMs) compared with bulk materials. In this case, antibacterial activities of MNPs/MOPNs against MDR bacteria have been reported by various investigations.^4^ ZnO and CuO NPs with less cytotoxicity compared to other MNPs/MONPs such as Ag NPs may be a suitable option to obtain efficient antibacterial formulation.^5,6^ Ions released by these MONPs are defined as a major antibacterial mechanism which can disrupt the integrity of cell envelope of bacteria.^7^ In addition, the antibacterial and biocompatibility of these NPs can be modified by several approaches. For instance, green synthesis of ZnO and CuO NPs by medicinal plant extracts can decrease cytotoxicity and increase therapeutic applications of these MONPs.^8,9^ For this purpose, there are many studies related to phytosynthesis of Ag, Cu, CuO, ZnO, and Fe_3_O_4_ NPs with antibacterial effects on sensitive and MDR bacteria.^3^ In this regard, green synthesized TiO_2_, Fe_3_O_4_, ZnO, Cu, and Ag NPs by *Artemisia haussknechtii* plant species demonstrated average diameter size of 92.58, 83.4, 60, 35.36 and 10.69 nm with antibacterial activities against Gram-positive and Gram-negative bacteria.^10-12^ In this study, we used *Mentha pulegium* plant species to synthesize ZnO and CuO NPs. *M. pulegium* is flowering plant in *Mentha* genus of Lamiaceae family which can be found widely in Middle East, North Africa and Europe regions as native species. Stems, leaves and flowers of this plant species have antiseptic activities against wide range of microorganisms.^13,14^ Result of disc diffusion assay for essential oils of *M. pulegium*against *E. coli* and *S. aureus* showed inhibition zone diameters (IZDs) by 12.6±0.5 and 21.4±0.8 mm respectively.^15^ In previous study, leaves extracts of *M. pulegium*were used to prepare AgNPs with size distribution in the range 5-50 nm. These MNPs demonstrated antibacterial effects on *E. coli*, *S. aureus* and *Streptococcus pyogenes* bacteria.^16^ In fact, both flower and leaf extracts of *M. pulegium*have shownantimicrobial properties.^17,18^ These abilities can be resulted from various oxygenated monoterpenes in secondary metabolites of this plant species.^15^

According to above argument, we used leaf/flower aqueous extract to fabricate ZnO and CuO NPs. After characterization of physicochemical properties of these MONPs by standard techniques, antibacterial activities of each MONP against antibiotic-resistant *E. coli* and *S. aureus* were determined by disc diffusion, agar well diffusion, minimum inhibition and bactericidal concentrations (MIC/MBC) assays. In the case of the bacterial loading on the glass surface, ATP-bioluminometer instrument was applied to measure cell number upon MONPs stress.

## Materials and Methods

### Materials

All materials obtained from commercial sources were utilized without further purification. Zinc nitrate [Zn(NO₃)₂ •6H_2_O], copper(II) sulfate (CuSO_4_), nutrient agar (NA), Mueller-Hinton agar (MHA), Mueller-Hinton broth (MHB) and peptone water (PW) are purchased from Sigma-Aldrich chemicals company (St. Louis, MO). Antibiotic discs were purchased from PADTAN TEB Company, Tehran, Iran.

### Preparation of Leaf/flower extract

Healthy leaves of *M. pulegium* were sampled from the Amrooleh mountainous region, in the Kermanshah province, west of Iran during July 2019 followed by identification and authentication by an expert of Kurdistan agriculture and resource research center (Sanandaj, Kurdistan). Aqueous leaf/flower extract of *M. pulegium* was obtained by freshly amassed leaf/flower (10 g). The leaves and flower surface were cleaned with running tap water, followed using distilled water, and air dried on a paper towel for 2 weeks. Dry leaves and flowers were grounded in a tissue grinder to get fine powder, and boiled with 150 mL of double distilled water at 60°C for 1 hour. The ﬁltered suspension was collected and stored at 4°C till further use.

### Phytosynthesis of CuO and ZnO NPs

In order to phytosynthesize CuO and ZnO NPs, the Erlenmeyer flask with 100 mL volume of CuSO_4_ and [Zn(NO₃)₂ •6H2O] salts by 0.1M concentrations were stirred for 3 hours at 25^○^C. The aqueous leaf/flower extract of *M. pulegium*was filtered via Whatman No. 1 filter paper followed by centrifugation at 5000 rpm for 1h. Twenty mL of resulted leaf/flower aqueous extract samples were separately added to 80 mL of CuSO_4_ and [Zn(NO₃)₂ •6H2O] at room temperatures under stirred condition for 24 hours. In order to purify NPs, mixture solutions were centrifuged at 4000 rpm for 30 minutes. After drying the colloidal solution, the resulting powder was crushed and subsequently the obtained sediment was washed repeatedly with absolute ethanol and deionized water. In the case of ZnO NPs synthesis, the samples were calcined at 400°C to obtain the powder for subsequent analyses.^19^

### Physicochemical characterization

UV-Vis spectroscopy (Tomas, UV 331), X-ray diffraction (XRD) analysis (model PW1730, PHILIPS, Netherlands), Fourier-transform infrared (FTIR) spectroscopy (AVATAR, Thermo, United States), and FE-SEM (MIRA III, TESCAN, Czechia) technique were applied to determine physicochemical properties of ZnO and CuONPs. Zeta potentials of each NP were indicated by DLS (model ZEN3600, MALVERN, United Kingdom). The intensities related to absorption peaks of ZnO and CuONPs were examined by UV-Vis spectroscopy in the wavelength range of 200 to 600 nm. XRD was applied in the scanning range of 10°-80°(2θ) using Cu Ka radiations of wavelength 1.5406 Å for identification of the crystal phases and determination of the average crystal size of NPs.

### Antibacterial activities

#### Bacteria species

*Escherichia coli* and *S. aureus*, as respectively gram-negative and gram-positive bacteria species were obtained from Kowsar hospital, Kurdistan University of Medical Sciences, Sanandaj, Iran. In order to determine the sensitivity of these species, after culturing of bacteria on NA medium and incubation for 24 hours at 37^○^C, antibiotics including amoxicillin, azithromycin, ciprofloxacin, cefixime, doxycycline, gentamycin, and sulfamethoxazole as the amount of 10 µg/disc were tested on these bacteria (Table 1).

**Table 1:** Disc diffusion (IZD (mm) ± SD) results for antibiotics sensitivity of *E. coli* and *S. aureus* (R=resistance). Values are averages of three independent analyses plus SD (n = 3)

**Antibiotic type**	***E. coli***	***S. aureus***
Amoxicillin	R	46.13 ± 1.02
Azithromycin	R	21.46 ± 0.5
Cefixime	R	19.9 ± 0.17
Ciprofloxacin	R	R
Doxycycline	R	R
Gentamycin	21.8 ± 0.72	40.33 ± 0.57
Sulfamethoxazole	R	R

### Agar well diffusion and MIC/MBC assays

Broth cultures of *S. aureus* and *E. coli* as cell density ≈ 1.5 × 10^8^ CFU/mL (standardized by 0.5 McFarland standard) were prepared in the PW medium with the concentration of 0.1%. The bacteria species were swabbed on the MHA plate. Wells (6 mm) were made with a sterile metal punch on the surface of the agar plates. Wells were filled by 50 µL of ZnO and CuONPs with serial concentrations of 0.625, 1.25, 2.5, 5, and 10 mg/mL followed by incubation at 37^○^C for 24 hours. IZDs were measured by calipers as averages of three independent analyses plus standard deviation.^20^ Minimum bacteriostatic and bactericidal concentrations of both NPs were indicated by MIC and MBC respectively. Firstly, the standard cell density of bacteria (0.5 McFarland) was cultured in 96-well microplate. Concentrations of NPs were varied via two-fold serial dilution (0.625, 1.25, 2.5, 5, and 10 mg/mL). The wells were monitored for turbidity as growth and non-turbidity as no growth following incubation of medium for period of 1 day at 37°C. 10 μL of the samples of each tube with no growth of bacteria were sub cultured onto the agar. The MIC results were indicated as the lowest concentration of the sample, which demonstrated clear fluid with no development of turbidity. Moreover, the MBC was determined as the highest dilution of each NP that did not generate a single bacterial colony on MHA after 1 day incubation period.^12^

### Statistical analysis

Statistical evaluating of results were performed by SPSS version16 software (SPSS Inc., Chicago, IL) and one way ANOVA (Tukey’s test) respectively. Results were presented in triplicates and averages plus standard errors were assessed as *P*≤0.05 of significant value.

### ATP-bioluminometer

ATP-bioluminometer (UltraSnap^TM^-Surface ATP) is used to determine bacterial loading on the environmental surfaces and medical equipment.^21,22^ For this purpose, NP with higher antibacterial activities was selected to measure bacterial removal ability from the infected surface. It should be considered that *E. coli* bacteria were more resistance to antibiotic compared to *S. aureus*. Four glass slides were sterilized in 150^○^C for 120 minutes followed by an addition of 100 µL of *E. coli* to each slide. The initial cell number of bacteria was measured as amount as 3673 by ATP-bioluminometer. Afterwards, volumes of 25, 50, and 100 µL of NPs with 5 mg/mL value were incubated on the surface of slides containing bacteria. One slide without NPs was considered as the control sample and cell number was obtained after 15 minutes.

## Results and Discussion

### UV-Vis spectroscopy and zeta potential

**Figure 1:**
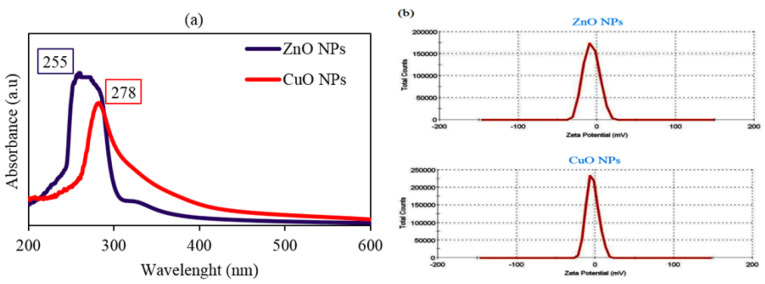
UV-Vis spectra (a) and zeta potential graphs (b) of green synthesized ZnO and CuO NPs with related peaks.

The formation of MONPs can be determined by their surface plasmon absorption band. UV-Vis spectra for ZnO and CuONPs showed peaks at 255 and 278 nm wavelengths respectively (Figure 1). In a similar study, there was a peak at 370 nm for green synthesized ZnO NPs by *Artemisia haussknechtii*, medicinal plant species.^19^ The maximum absorbance for biosynthesized CuONPs via *Malus domestica* leaf extract was at 335 nm wavelength.^23^ The absorption band at 366 nm was found for green prepared ZnONPs via *Catharanthus roseus* leaf extract.^24^ Electrokinetic potentials of both NPs in colloidal dispersion were indicated by zeta potential results. ZnONP demonstrated -7.14 mV and 4.96 mS/cm for zeta potential and conductivity respectively. Also, the values of zeta potential and conductivity were respectively -3.45 mV and 3.43 mS/cm for CuONPs. In contrast, the positive charge of zeta potential as the amount of 2.61 mV was found for phytosynthesized ZnONPs using* Solanum torvum* leaf extract.^25^ Similarly, there was a negative charge of zeta potential around -30 mV at pH≈7 for green synthesized CuONPs by leaf extract of* Punica granatum*plant species.^26^

### XRD results

**Figure 2:**
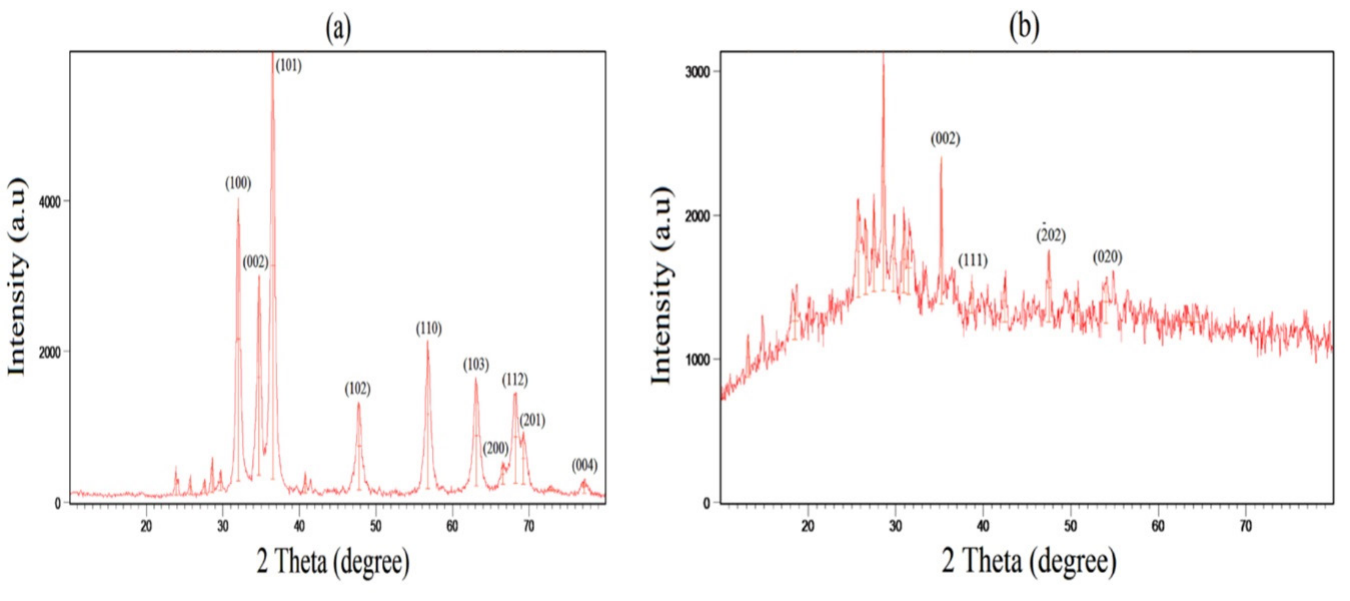
XRD patterns of green synthesized ZnO NPs (a) and CuO NPs (b).

As shown in Figure 2a and 2b, XRD analysis illustrated crystalline structures and phases of ZnO and CuONPs. The sharp peaks at (100), (002), (101), (102), (110), (103) and (112) were corresponded to 2θ values of 32^○^, 34.69^○^, 36.49^○^, 47.79^○^, 56.78^○^, 63.09^○^ and 68.24^○^ respectively (Figure 2a). Similar diffraction lattice planes by the hexagonal wurtzite structure were observed at 31.46^○^, 34.29^○^, 36.33^○^, 47.51^○^, 56.50^○^, 62.84^○^ and 67.79^○^ for green synthesized ZnONPs via *Laurus nobilis* leaves aqueous extract.^27^ As illustrated in Figure 2b, in the case of CuONPs, there was a crystallite structure of face-centered cubic structure (FCC) by the peaks at 35.20^○^, 38.66^○^, 47.50^○^ and 53.99^○^ for the planes of (002), (111), (-202) and (020) respectively.^28^ These planes were indicated for prepared CuONPs by aqueous extract of oak fruit hull.^29^ The obtained crystal size of ZnONPs and CuONPs were respectively 18.09 and 18 nm. Hexagonal morphology of ZnO NPs with 36.83 nm grain size was observed in the case of phytofabricated ZnONPs via *C. roseus* plant leaf extract.^24^ Phytosynthesized ZnONPs by *A. haussknechtii* leaf aqueous extract showed an average crystal size of 53 nm.^19^ In addition, CuONPs biosynthesized by *Halomonaselongata* IBRC-M 10214 demonstrated grain size in the range of 57-79 nm.^30^ According to the present results, grain sizes of our NPs were smaller compared to similar previous investigations.

### FTIR spectra

**Figure 3:**
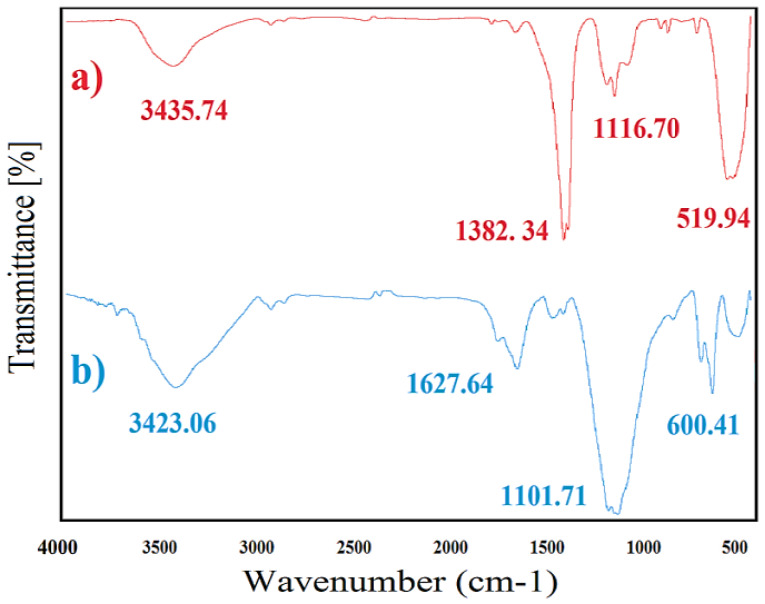
FTIR spectra related to phytosynthesized ZnONPs (a) and CuONPs (b).

FTIR spectra of ZnO and CuONPs are presented in Figure 3a and 2b. Prominent peaks at 3435.74 and 3423.06 cm-^1^ were related to -OH stretching vibration, which can be associated to water adsorption on NPs surface. In the case of ZnONPs, peaks at 1382.34, 1116.70 and 519.94 cm-^1^ wavenumber were corresponded to C-H bending (aldehyde and alkane), C-O stretching (tertiary alcohol and aliphatic ether) and C-I stretching (halo compound) respectively (Figure 3a). For CuONPs, sharp and intense peaks were at 1627.64, 1101.71 and 600.41 cm-^1^ respectively related to C=C stretching (alkene), C-O stretching (secondary alcohol and aliphatic ether) and C-Br stretching (halo compound) (Figure 3b). Similar peaks also were indicated to green synthesized ZnO and CuONPs by *Abelmoschus esculentus* mucilage and algal extract respectively.^31,32^ These functional groups may be resulted from the interaction of MONPs with primary and secondary metabolites of leaf extract.^33^ C-O stretching (secondary alcohol and aliphatic ether) and C-O stretching (tertiary alcohol and aliphatic ether) were a common functional group for both CuO and ZnONPs. This functional group can be resulted from the presence of secondary metabolites containing alcohol and aliphatic ether in leaf/flower extract.^34^ In addition, these peaks may be responsible for the stabilization of ZnO and CuONPs.^35^

### SEM and EDX analyses

**Figure 4:**
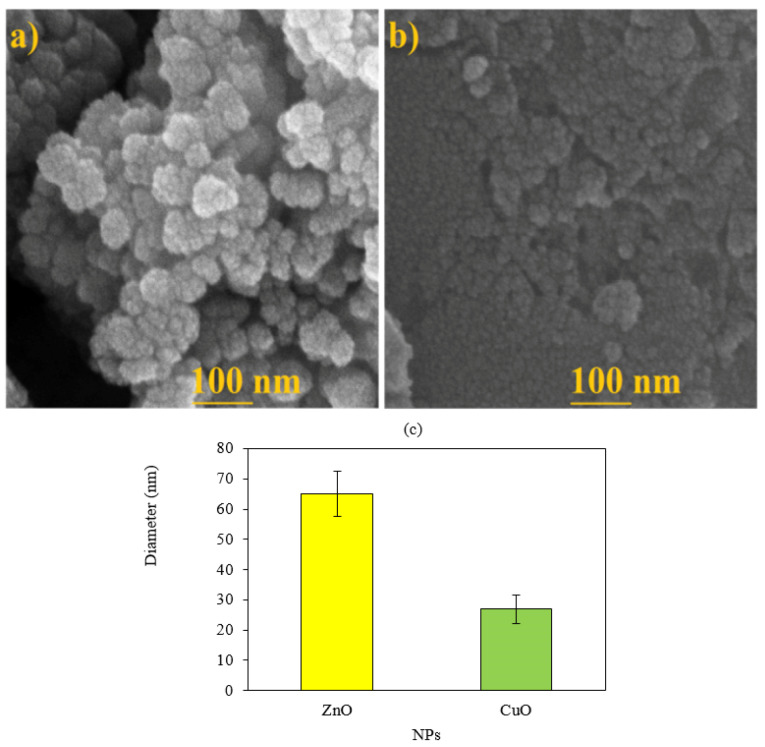
SEM images of ZnO NPs (a) and CuO NPs (b). Average diameter sizes of each NP (c)

**Figure 5:**
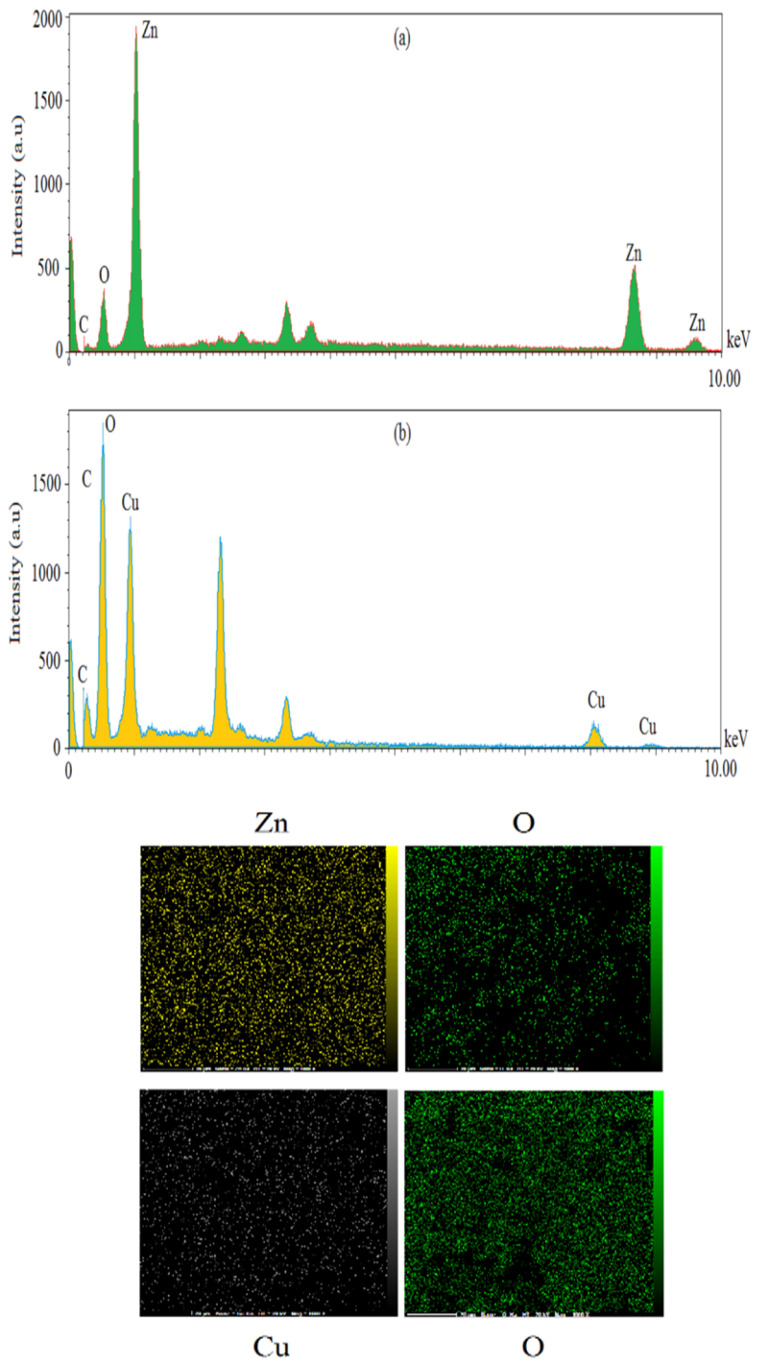
EDX spectra of green synthesized ZnO NPs (a) and CuO NPs (b) with related elemental maps.

According to the results of SEM images, spherical shape was a common shape of ZnO and CuO NPs (Figure 4a and 4b). Average diameter sizes for ZnO and CuO NPs were respectively 65.02±7.55 and 26.92±4.7 nm (Figure 4c). As shown in Figure 5, EDX analysis of ZnONPs showed respectively 77.98%, 12.25%, 4.85%, and 3.05% for Zn, O, C and K elements (Figure 5a). As illustrated in Figure 5b, elemental weights of CuONPs were 51.39%, 21.49%, 13.91%, 9.91% and 3.31% for O, C, Cu, S and K respectively. Oxygen, carbon and potassium elements were common in green synthesis of these MONPs by *M. pulegium*. Contribution of carbon, phosphor and sulfur indicated for green synthesized CuONPs using *Malus domestica* leaf extract.^23^ In addition to zinc and oxygen, lichen synthesized ZnONPs by *P. muralis* showed sulfur and chlorine elements in the EDX spectrum.^36^

### Agar well diffusion and MIC/MBC assays

**Figure 6:**
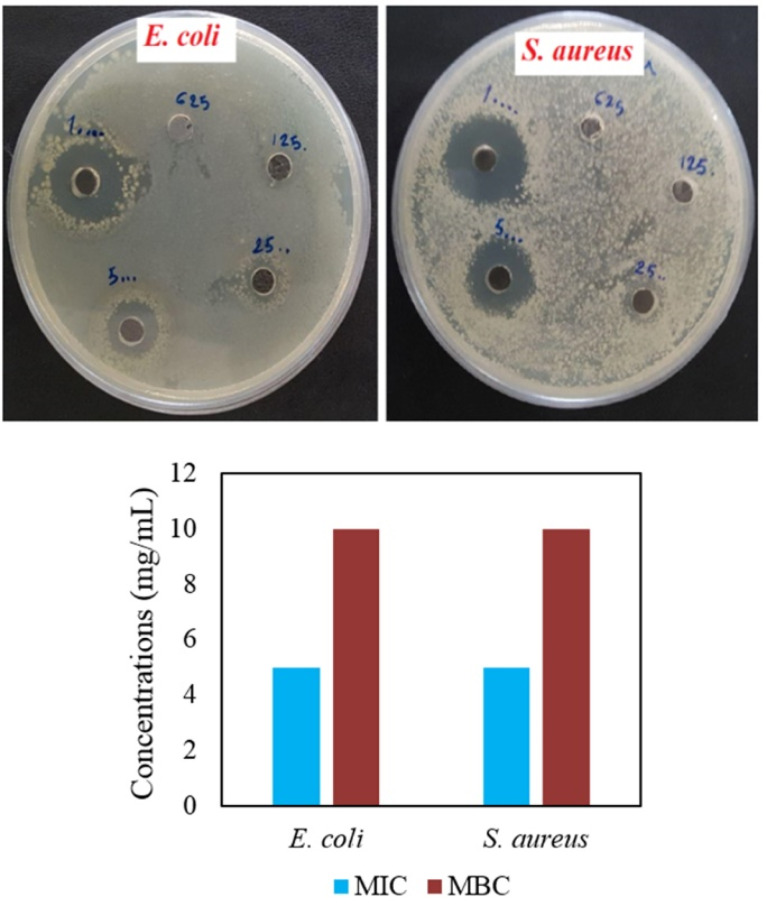
Images showing IZDs for *E. coli* and *S. aureus* bacteria at serial two-fold dilution (625, 1250, 2500, 5000, and 10000 µg/mL) as well as MIC and MBC results.

Agar well diffusion showed antibacterial activities of CuONPs contrast to ZnONPs with any antibacterial effects in all concentrations (Table 2 and Figure 6). At lower amounts of CuONPs (0.625 and 1.25 mg/mL), both bacteria showed resistance. In a comparative way, *S. aureus* demonstrated higher sensitivity than *E. coli*. At a higher concentration (10 mg/mL), IZD values of 17.56±0.4 and 20.96±0.45 mm were observed for *E. coli* and *S. aureus* respectively (Table 2). In the previous study, streptomycin antibiotic and phytosynthesized Cu/Cu_2_O NPs via *Stachys lavandulifolia* flowers aqueous extract showed 14 and 12 mm IZDs in the case of *Pseudomonas aeruginosa* bacteria.^37^ Green synthesized CuONPs with 22-25 nm crystallite size via mint leaf extract showed 38 and 35 mm IZDs for *Bacillus subtilis* and *E. coli* bacteria respectively.^38^ Biosynthesized CuONPs by *Bacillus* sp. FU4 demonstrated IZD of 33±0.57 mm toward *E. coli* ATCC 25922.^39^ There were IZD values by 10 and 11 mm for respectively *E. coli* and *S. aureus* bacteria upon biosynthesized CuONPs via *Halomonaselongata* IBRC-M 10214.^30^ Prepared CuONPs by different methods involving chemical precipitation, microwave irradiation, and hydrothermal methods showed respectively 27 mm, 25 mm, and 22 mm IZDs against *S. aureus.*^40^ For both bacteria, MIC and MBC assays illustrated respectively 5 and 10 mg/mL concentrations. MIC and MBC amount for synthetic CuONPs (with a diameter of 48 ± 7 nm) was >100 µg/mL toward *Salmonella Typhimurium*bacteria.^41^

**Table 2:** Results of agar well diffusion (IZD (mm) ± SD) for CuONPs against *E.coli* and *S. aureus* bacteria

** Concentration (mg/mL)**	***E. coli ***	***S. aureus***
0.625	-	-
1.25	-	-
2.5	6.46 ± 0.152	7.66 ± 0.57
5	14 ± 0.5	16.5 ± 0.5
10	17.56 ± 0.4	20.96 ± 0.45

### ATP-bioluminometer

Growth of pathogenic microorganism particularly MDR bacteria on the hospital surfaces is a complicated issue, which should be removed to obtain a sterilized environment. Results of ATP-bioluminometer test illustrated the bactericidal activity of CuONPs at 50 and 100 µL compared to control and 25 µL of NPs (Table 3). The present study showed prominent antibacterial activities of phytosynthesized CuONPs by leaf/flower aqueous extract of *M. pulegium* medicinal plant. These findings can be resulted from antibacterial capacities related to plant metabolites as reducer/stabilizer agents and the ability of CuONPs to produce reactive oxygen species (ROS) in bacterial medium. In contrast to ZnONPs, the interaction of CuONPs with metabolites of *M. pulegium* led to synergism antibacterial activities. As illustrated in previous reports, hydrogen peroxide (H_2_O_2_), hydroxyl (^*^OH), superoxide (^*^O_2_-) and peroxide (^*^O_2_-^2^) are common ROS, which may be resulted in damage of bacterial envelope and biological macromolecules.^28,42,43^

**Table 3:** Antibacterial results of CuONPs on the glass slide after 15 minutes incubation

**CuONPs volume (µL)**	25	50	100	Control
**Bacteria number**	368	0	0	2218

## Conclusion

This study reported a one-pot, cost-effective, and eco-friendly method to synthesize ZnONPs and CuONPs by leaf/flower extract of *M. pulegium*medicinal plant. The contribution of various metabolites in the stabilizing of MONPs was confirmed by FTIR spectra. C-O stretching (tertiary alcohol and aliphatic ether) and C-O stretching (secondary alcohol and aliphatic ether) were a common functional group for both ZnO and CuONPs. This functional group can be related to secondary metabolites containing alcohol and aliphatic ether with MONPs stabilization property. Based on the results of XRD spectra, the hexagonal wurtzite and FCC crystallite structures were determined for ZnO and CuO NPs respectively. Despite the similar shape (spherical) for both MONPs, there was a smaller size of CuONPs compared to ZnONPs. Antibacterial effects on antibiotic-resistant *E. coli* and *S. aureus* were higher for CuONPs in the face of ZnONPs. A large surface area as well as more reactivity of CuONPs rather than ZnONPs may be resulted in more ROS formation in the bacterial medium. The structural difference in the cell envelope of Gram-positive (cell wall and cell membrane) and Gram-negative (outer cell membrane, cell wall, and inner cell membrane) bacteria can impact on antibacterial capacities of MONPs. In the nutshell, *M. pulegium* medicinal plant can be a considerable option to ecofriendly phytosynthesize CuONPs with significant antibacterial activities toward pathogenic bacteria with antibiotic resistance property.

## Ethical Issues

Not applicable.

## Conflict of Interest

The authors declared no conflict of interest.

## Acknowledgments

This article was extracted from a project supported by Kurdistan University of Medical Sciences (IR.MUK.REC. 1397/338). Hereby, we would like to express our gratitude to the sponsors of this study.
